# Historical Insights Into the Purification of Polyphenol Oxidase From Plants

**DOI:** 10.1002/fsn3.71175

**Published:** 2025-11-21

**Authors:** Cesar G. Vazquez‐Lima, Mariana Quintana‐Quirino, Ana Luisa Bravo, Roxana López‐Simeon, Hugo Nájera

**Affiliations:** ^1^ Posgrado en Ciencias Naturales e Ingeniería Universidad Autónoma Metropolitana ‐ Cuajimalpa Ciudad de México México; ^2^ Departamento de Ciencias Naturales Universidad Autónoma Metropolitana ‐ Cuajimalpa Ciudad de México México

**Keywords:** enzymatic browning, fruits, plants, polyphenol oxidase, purification

## Abstract

Over the past 50 years, different methods have been developed to purify polyphenol oxidase (PPO), a key enzyme involved in the oxidation of phenolic compounds in plants. This review presents a comprehensive summary of the advances in purification techniques, highlighting the evolution of the methods used over the years to achieve partially purified or pure enzymes, while also discussing their advantages and limitations. Furthermore, this review examines the biochemical properties of PPO that are most significant for its potential biotechnological applications. A comparison of the amino acid sequences and tertiary structures of crystallized PPO is also included, enabling the identification of structural similarities among PPO from different species. Finally, this review has been written as a guide for decision‐making regarding the selection of an appropriate PPO purification strategy, taking into account the required level of purification and the enzyme's source.

## Graphical abstract

Overview of purification methods for polyphenol oxidase. Advancements in achieving partial or homogeneous purification. Key biochemical properties relevant to polyphenol oxidase.

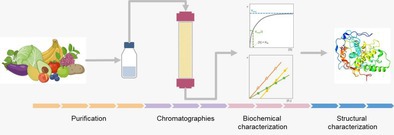

**Abbreviations**
AC: affinity chromatography
ATPS: aqueous two‐phase system
HHP: high hydrostatic pressure
HIC: hydrophobic interaction chromatography
IEC: ion exchange chromatography
PEG: polyethylene glycol
PMSF: phenylmethylsulfonyl fluoride
PPO: polyphenol oxidase
PVPP: polyvinylpolypyrrolidone
SEC: size exclusion chromatography
TIPS: temperature‐induced phase separation
TPP: triphasic partitioning

## 1 Introduction

For nearly 50 years, the enzyme polyphenol oxidase (PPO) has been studied in arthropods, fungi, and plants, especially in fruits. Interest in PPO is primarily related to the quality of food, specifically fruits, as the action of PPOs may contribute to a decrease in nutritional value. PPO has a binuclear copper center and, in principle, performs two activities. The first one, known as monophenolase activity, is based on the insertion of oxygen in a position ortho to an existing hydroxyl group in an aromatic ring. The second activity is known as catechol oxidase, and is based on the oxidation of diphenol, resulting in quinones (Mayer 2006). The polymerization of the formed quinones results in a phenomenon known as enzymatic browning.

Polyphenol oxidases are classified into two main groups: tyrosinases (EC 1.14.18.1), which have monophenolase and catechol oxidase activity, and catechol oxidases (EC 1.10.3.1), which were previously thought to lack monophenolase activity but do have it for a specific substrate. Sometimes, PPO is confused with other oxidase enzymes, like laccases. Laccases have a trinuclear center, targeting a wide range of diphenols (Boeckx et al. 2015).

PPO is found mainly in the thylakoid membranes and other non‐green vesicles. Phenolic compounds are initially restricted in vacuoles and are therefore far from interacting with PPO (Mayer and Harel 1979). Given the notorious remoteness of the enzyme from these compounds, it has been proposed that the interaction of the enzyme with the substrate requires the destruction of cellular compartments, which is achieved through mechanical damage, such as a fall or injury (Queiroz et al. 2008).

Polyphenol oxidase has been extensively studied over the years, primarily due to its significance in determining the quality of fruits and vegetables. For this reason, purifying PPO has been a goal for several years, but has been challenging to achieve.

Inhibition of PPO to prevent enzymatic browning and thus preserve the nutritional value has been investigated through various methods. Most approaches include physical and chemical processes, such as B‐cyclodextrin (Singh et al. 2015), chitosan (Xing et al. 2011), thermosonication (Anaya‐Esparza et al. 2017), ultrasound, ascorbic acid (Jang and Moon 2011), and microwave (Palma‐Orozco et al. 2012), among others, which will be discussed later. Non‐thermal treatments, such as high hydrostatic pressure (HHP) processing (Tinello and Lante 2018) and the use of cold plasma (Illera et al. 2019), have also emerged to inhibit PPO.

In addition to its role in enzymatic browning, PPO also has other functions. One of its notable features is its involvement in herbivore‐associated defense, an idea initially proposed by Felton et al. (1989). A review was recently published analyzing the studies that were done with plants, with the authors concluding that these studies were limited in terms of research systems and that the defensive role of PPO on insect attack is complex in different experimental conditions; it is therefore essential to conduct further research to clarify the mechanism by which PPO participates in herbivore‐associated defense (Zhang and Sun 2021).

Regarding structural studies, it has been reported that all plant PPOs have an N‐terminal domain, a central section, and a C‐terminal domain. The N‐terminal domain is responsible for determining the cellular location of the enzyme; these locations can be the thylakoid or the vacuole (Kaintz et al. 2015). The central section consists of two tyrosinase domains, which are protected by the C‐terminal domain (Pretzler and Rompel 2024). The entire central section and the C‐terminal domain are collectively referred to as the latent enzyme. For the polyphenol enzyme to be active, the C‐terminal domain must be cleaved during the maturation process. Latent enzymes in vitro can be activated by detergents (such as SDS), acidic pH, or fatty acids (Molitor et al. 2015).

In contrast, in vivo activation can occur through the interaction of the enzyme with its substrate (Boeckx et al. 2015). Although the enzyme has been studied for a long time, there are few crystallographic structures; therefore, this review will focus primarily on those from plants. Crystallization of the enzyme has been achieved from various organisms, such as the bacterium *Streptomyces castaneoglobisporus* (PDB 1WX2), the fungus *Agaricus bisporus* (PDB 2Y9W), and the arthropod *Limulus polyphemus* (PDB 1JS8).

Regarding applications, PPO has been used in various fields, including the food industry, where it has developed additives that enhance product quality (Quan et al. 2019), biosensors for phenol identification (Li et al. 2010; Raymundo‐Pereira et al. 2020; Sartori et al. 2011), and devices for immobilizing or eliminating phenolic compounds (Altinkaynak et al. 2018; Gür et al. 2019; Mukherjee et al. 2013).

This review examines the findings from the past 50 years of the PPO study, intending to identify the optimal purification train required to achieve a homogeneous enzyme.

## 2 Purification

**FIGURE 1:**
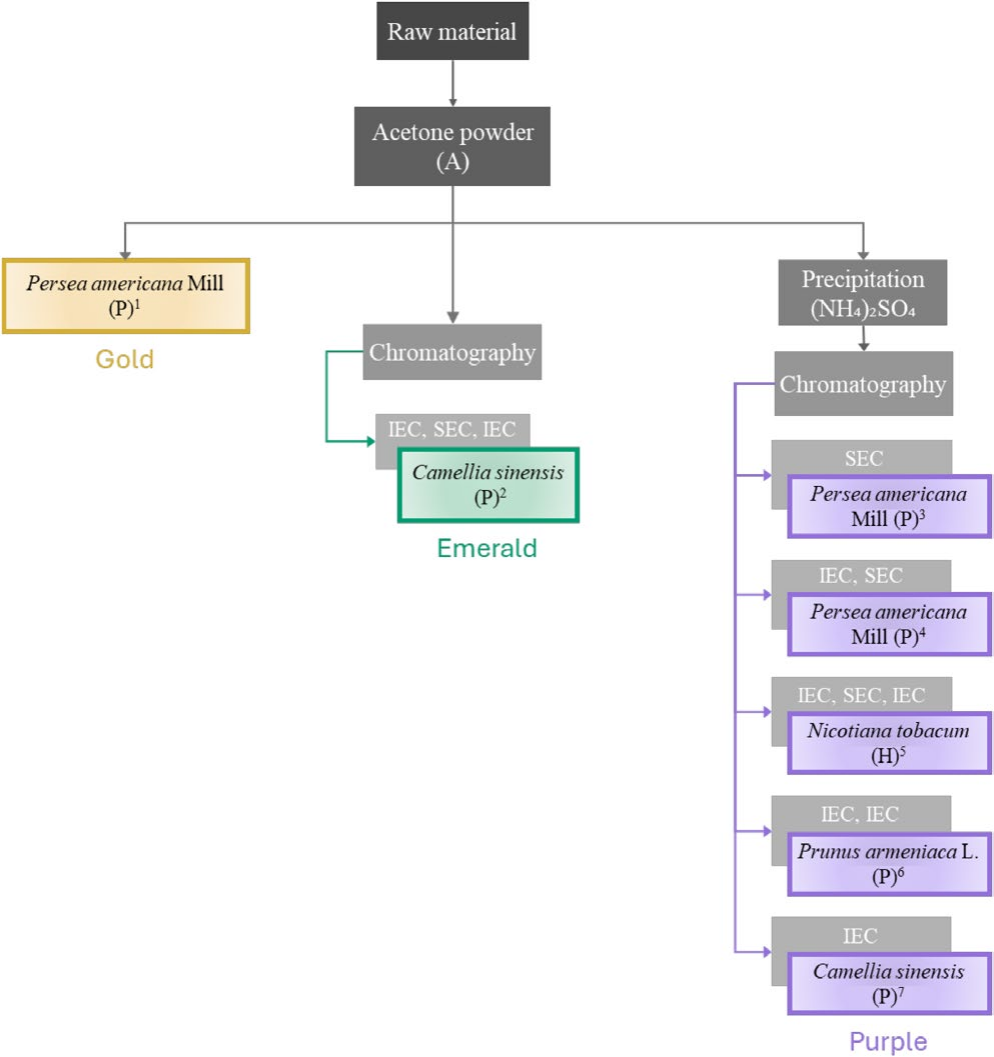
Purification trains with acetone. Gold *Persea americana* ^1^ (Gómez‐López 2002; crude extract). Emerald, *Camellia sinensis* ^2^ (Halder et al. 1998; IEC, DEAE cellulose column preequilibrated with 50 mM K phosphate buffer pH 7.0, SEC, Sephacryl S‐200 column preequilibrated with 50 mM K phosphate buffer, pH 7.0, IEC, hydroxyapatite 10 mM Na phosphate buffer, pH 6.8). Purple, *Persea americana* Mill^3^ (Dizik and Kanapp 1970; 50%–70% ammonium sulfate, SEC Sephadex G‐150 preequilibrated with McIlvaine buffer, pH 6.8); *Persea americana* Mill^4^ (Kahn 1976; 40%–75% ammonium sulfate), IEC DEAE‐cellulose, preequilibrated with 50 mM Na phosphate buffer pH 6.5 and SEC Sephadex G‐100 preequilibrated with 0.05 M Na phosphate buffer pH 6.5; *Nicotiana tobacum* ^5^ (Shi et al. 2002; 30%–80% of ammonium sulfate), IEC DEAE–Sephadex A‐50 column preequilibrated with 0.05 M Tris–HCl buffer, pH 7.5, SEC Sephadex G‐75 column preequilibrated with 50 mM Na phosphate buffer pH 6.5, IEC CM‐ Sephadex C‐50 preequilibrated with 50 mM Na phosphate buffer, pH 6.5; *Prunus armeniaca* ^6^ (Derardja et al. 2017; 0%–85% ammonium sulfate), IEC Q‐Sepharose FF preequilibrated with 10 mM Tris–HCl, pH 8, IEC Mono S HR 5/50 GL preequilibrated with 10 mM sodium acetate buffer, pH 5. *Camellia sinensis* ^7^ (Teng et al. 2017; 0%–80% ammonium sulfate), Q‐Sepharaose Fast Flow equilibrated with 1 L of 0.02 mol/L Tris–HCl buffer (pH 7.5) containing 100 mL/L glycerol; Sephadex G‐75, equilibrated with 0.02 mol/L Tris–HCl buffer (pH 7.5) containing 100 mL/L glycerol and 0.1 mol/L NaCl.

**FIGURE 2:**
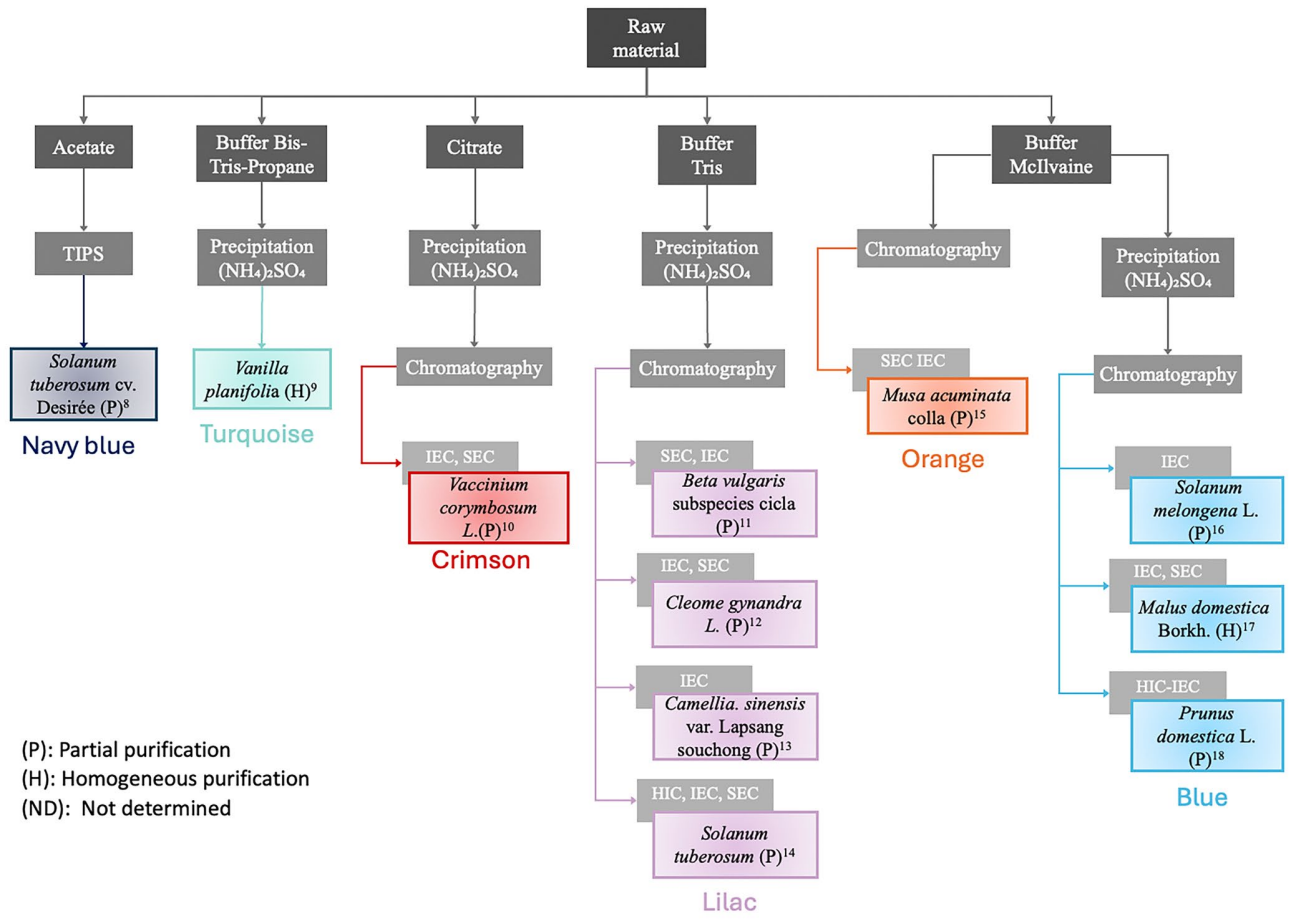
Purification using different buffer types. Navy blue *Solanum tuberosum cv. Desirb* ^8^ (Sánchez‐Ferrer et al. 1993; TIPS). Turquoise *Vanilla planifolia* ^9^ (Waliszewski et al. 2009; 30%–80% ammonium sulfate). Crimson *Vaccinium corymbosum* L.^10^ (Wei et al. 2021; 40%–90% ammonium sulfate), IEC, DEAE‐Sepharose preequilibrated with 0.02 M TrisHCl buffer pH 7.4, SEC Sephadex G‐150 preequilibrated with 0.02 M Tris–HCl buffer pH 7.4 containing 0.15 M NaCl. Lilac, *Beta vulgaris* Subspecies Cicla^11^ (Gao et al. 2009; 20%–80% ammonium sulfate), SEC Sephadex G‐75 preequilibrated with 20 mM Tris–HCl buffer pH 7.5, IEC DEAE‐Sepharose preequilibrated with 20 mM Tris–HCl buffer pH 7.5; *Cleome gynandra* ^12^ (Gao et al. 2011; 40%–80% ammonium sulfate), IEC DEAE‐Sepharose preequilibrated with 20 mM Tris–HCl buffer pH 7.5, SEC Sephadex G‐75 preequilibrated with 20 mM Tris–HCl buffer pH 7.5, *Camellia sinensis* ^13^ (Ke et al. 2021; IEC, UNOsphere Q anion exchange preequilibrated with 20 mM Tris–HCl pH 9.0 containing 5 M urea); *Solanum tuberosum* ^14^ (Liu et al. 2023; 35%–55% ammonium sulfate), IEC DEAE Sepharose column equilibrated with 20 mM Tris–HCl pH 7.2, HIC phenyl Sepharose column pre‐equilibrated with 20 mM Tris–HCl pH 7.2 and SEC Superdex 75 equilibrated with 10 mM phosphate buffer pH 6.0, 150 mM NaCl. Orange *Musa acuminata* Colla^15^ (Ngalani et al. 1993; SEC Sephadex G 100 preequilibrated with 0.1 M McIlvaine buffer, pH 7.0, IEC a DEAE‐cellulose DE 32 preequilibrated with 0.1 M McIlvaine buffer, pH 7.5). Blue *Solanum melongena* L.^16^ (Roudsari et al. 1981; 30%–70% ammonium sulfate, SEC Sephadex G 25 preequilibrated with 5 mM McIlvaine buffer, pH 7.5, HIC DEAE cellulose preequilibrated with 25 mM McIlvaine buffer, pH 7.5); *Malus domestica* ^17^ (Han et al. 2020; 50%–80% ammonium sulfate, IEC DEAE‐Sepharose preequilibrated with 0.05 M phosphate buffer pH 6.80, SEC Sephacryl S‐200 HR 16/60 preequilibrated with 0.05 M phosphate buffer, pH 6.8 containing 0.15 M NaCl); *Prunus domestica* L.^18^ (Ioniţă et al. 2017; 30%–90% ammonium sulfate, HIC HiPrep Phenyl FF 16/10 preequilibrated with 20 mM phosphate buffer pH 7.5 and 1.0 M ammonium sulfate, IEC preequilibrated with Mono Q 5/50 GL 20 mM phosphate buffer pH 7.5). ND, not determined.

This review outlines the various paths that can be taken to carry out PPO purification, starting from crude extract (in some cases, the final step involves crude extract) and leading to partial or complete purification (homogeneity). To achieve a better understanding of all purifications in plants carried out since 1970, a schematic map is shown to illustrate the methods for polyphenol oxidase purification. There are two main paths; in the first, the crude extract is prepared with acetone powder, whereas the second uses lysis buffers (Figures 1 and 2).

During the 1970s, acetone powder was used to obtain the extract, which eliminated most of the interfering compounds. Since the 1980s, various buffers have been used more frequently, and both the yield and purification fold have been consistently calculated.

The following section will discuss the different purification paths. The colors mentioned refer to different purification trains in the corresponding figures.

### 2.1 Acetone Powder‐Based Trains

There are three purification trains for acetone powder. Acetone precipitation is a beneficial technique for protein purification, sample concentration, and cost‐effectiveness, making it a valuable tool in proteomics and other applications (Feist and Hummon 2015). It effectively removes impurities such as salts and detergents while concentrating protein samples. The method is affordable and straightforward due to the availability of reagents. When optimized with salts and under appropriate conditions, high protein recovery rates can be achieved, making it suitable for downstream processes such as 2‐D electrophoresis, SDS‐PAGE, and protein assays (Niu et al. 2018; Wongpia et al. 2015).

However, there are some drawbacks, including the risk of protein denaturation, which can complicate the re‐dissolution of the protein pellet. For this reason, it is often necessary to use a low temperature to avoid this process. A single precipitation might not eliminate all contaminants, often necessitating multiple rounds of precipitation. Additionally, the efficiency of protein recovery can vary depending on the specific sample, necessitating careful optimization of factors such as acetone concentration, salt presence, and initial protein levels (Thermo 2009).

The first purification train (gold) involves the preparation of a crude extract, followed by experiments. For instance, this method has allowed the study of some biochemical properties of two varieties of avocado, *Persea americana* Mill (Gómez‐López 2002). This study aimed to perform biochemical tests on crude extracts to obtain valuable information that would guide the development of food products. For this reason, it was not necessary to purify PPO to homogeneity.

Another train (emerald) involves a purification process by chromatography after obtaining a crude extract, as demonstrated in the case of *Camellia sinensis* (Halder et al. 1998). Here, a partial purification was achieved with a 314‐fold purity. The authors propose a series of chromatographic steps consisting of ion exchange chromatography (IEC), size exclusion chromatography (SEC), and IEC to purify the enzyme and also use a substrate staining technique to demonstrate the presence of at least three isoforms. This thorough process enabled them to achieve homogeneous PPO purification.

Another option (purple) includes a crude extract precipitation step with ammonium sulfate, followed by chromatography as a final step. One example of this process is *P. americana* Mill. (Dizik and Kanapp 1970), *Prunus armeniaca* L. (Derardja et al. 2017), and *Nicotiana tabacum* (Shi et al. 2002), having a fold purification of 28, 23.2, and 100, respectively (purple). In the case of *Pr. armeniaca* L., it is mentioned that polyphenol oxidase (PPO) from this species was previously partially purified. Therefore, the authors undertook the task of finding a method that would allow them to purify the PPO in its latent form, which they suggest will facilitate the development of a better inhibition method for enzymatic browning in this fruit.

Regarding the study of *N. tabacum* , a purification method has been established to isolate polyphenol oxidase, focusing on IEC, SEC, and IEC. Achieving this purification enabled the researchers to determine the molecular weight of the enzyme and its biochemical parameters.

In summary, the purification of polyphenol oxidase from the specified fruits can be tailored to meet the specific research objectives. Partial purification may suffice if the goal is to study the enzyme's basic properties and functions. However, complete purification is likely necessary for detailed mechanistic studies or applications in food technology or pharmaceuticals. This flexibility in purification strategy allows researchers to optimize their approaches based on the intended outcomes, ultimately enhancing the understanding and utilization of polyphenol oxidase in various fields.

Ammonium sulfate precipitation is one of the most widely used methods in protein fractionation, particularly during the initial stages of purification from crude extracts. Its principle is that high salt concentrations can compete with proteins for water molecules, thereby reducing the availability of solvent. This competition increases surface tension and promotes hydrophobic interactions between proteins, leading to their aggregation and subsequent precipitation (Zou et al. 2024).

Although various salts can be used as precipitating agents, ammonium sulfate offers particularly advantageous characteristics: it is highly soluble in water, and at saturation, its molarity is high enough to precipitate most proteins. It is an inexpensive compound, readily available in pure form, and it also contributes to stabilizing the structure of many proteins during the process (Burgess 2009; Englard and Seifter 1990).

The use of ammonium sulfate allows for the removal of a significant proportion of non‐protein impurities and the concentration of the target protein (Zou et al. 2024). Among the various available methodologies, the most common approach involves the direct addition of solid ammonium sulfate to the protein extract until a specific saturation percentage is achieved (Burgess 2009). This strategy is practical, reproducible, and suitable for both laboratory and industrial‐scale applications.

However, the use of high concentrations of this salt may pose some disadvantages, since as the salt concentration increases from one step to another, the achieved purification may decrease compared to the previous fraction (Englard and Seifter 1990; Nadar et al. 2017). In some cases, conformational changes may be induced in the proteins that do not necessarily compromise their structural stability and, in solution, may protect most proteins from denaturation (Panadare and Rathod 2018).

Therefore, it is essential to have prior knowledge of the solubility of the protein of interest, as the critical salt saturation required for its precipitation depends on multiple factors, including the biological source, the pH of the medium, and the temperature of the solution (Nadar et al. 2017).

### 2.2 Buffer‐Based Trains

The commonly used buffers for PPO purification are Acetate (pH 3.6–5.6), McIlvaine (pH 2.0–8.0), sodium phosphate (pH 5.8–8.0), Tris (pH 7.0–9.0), and Bis‐tris propane (pH 6.0–9.5). These buffers differ in their pH ranges, but all of them are considered to be close to neutral pH. Higher or lower pH levels were tested occasionally, but provoked inhibition or were studied for PPO stability.

Figure 2 shows the first purification train (navy blue) using acetate buffer. Interestingly, this pathway uses a temperature‐induced phase separation (TIPS) process. A two‐phase separation system was created using Triton X‐114 to purify PPO from *Solanum tuberosum* cv Desirée partially. The reason for proposing this separation system is that they sought a new purification method for the PPO found in this species to study the enzyme's kinetic characteristics. Their results showed that using the detergent Triton X‐114 helped remove the phenols from the tubers of *S. tuberosum* cv. Desirée, preventing enzymatic browning during purification. This eliminates the need for acetone, which can inhibit the enzyme. This method only worked for isolating the soluble enzyme, as the enzyme's hydrophobic forms remained in the detergent‐rich phase (Sánchez‐Ferrer et al. 1993).

Only one assay (turquoise) has been reported for using Bis‐Tris Propane Buffer, in which the precipitation of crude extract from *Vanilla planifolia* was the final step (Waliszewski et al. 2009; Figure 2). The goal was to extract, purify, and characterize the PPO from vanilla beans to determine the kinetic parameters, optimal reaction conditions (pH and temperature), thermal stability, and inhibition of enzymatic activity. It has been observed that using Bis‐Tris Propane buffer provides better removal of impurities and higher product purity, opening up intriguing possibilities for its application in PPO extraction and purification (Chen et al. 2019).

Three PPO isoforms were purified from *Vaccinium corymbosum* L. with molecular weights (MW) of 36 kDa (PPO‐3 and PPO‐4), and a two‐subunit isoform with MWs of 36 and 68 kDa (PPO‐2), using a combination of citrate buffer, ammonium sulfate precipitation, IEC, and SEC (Wei et al. 2021; Figure 2, Crimson). This study aimed to propose a new methodology that would enable the authors to successfully purify PPO from *Vaccinium corymbosum* L., a process that had not been achieved previously. In addition to purifying the isoenzymes found in this species, they also determined the kinetic parameters of each one.

When the Tris buffer was used (lilac), the crude extract precipitation was made with ammonium sulfate, followed by one or two chromatographies (Figure 2). In every species, the PPO purification was partial (Gao et al. 2009, 2011; Ke et al. 2021). As shown in Figure 2, the only enzyme successfully purified homogeneously was the PPO from *S. tuberosum* , so this approach should be considered for studies on the enzyme from this species.

Two different options are reported when using McIlvaine buffer (Figure 2). The orange train performs chromatography after the crude extract is recovered. Ngalani et al. (1993) obtained an 8‐fold PPO purification from *Musa acuminata* Colla following this method. Although chromatography was used in this study to purify the PPO, it was not obtained in a homogeneous form. It is important to note that this methodology was employed to discover how to prevent enzymatic browning during the processing of plantain puree.

Another possibility (blue) consists of the crude extract precipitation followed by chromatography (Ioniţă et al. 2017; Ngalani et al. 1993; Yue‐Ming et al. 1997). This method purified PPO from *Malus domestica* Borkh (Han et al. 2020) to homogeneity with a 21‐fold purification. If the study and purification of this apple species are required, this purification method will allow the PPO to be obtained in its pure form.

**FIGURE 3:**
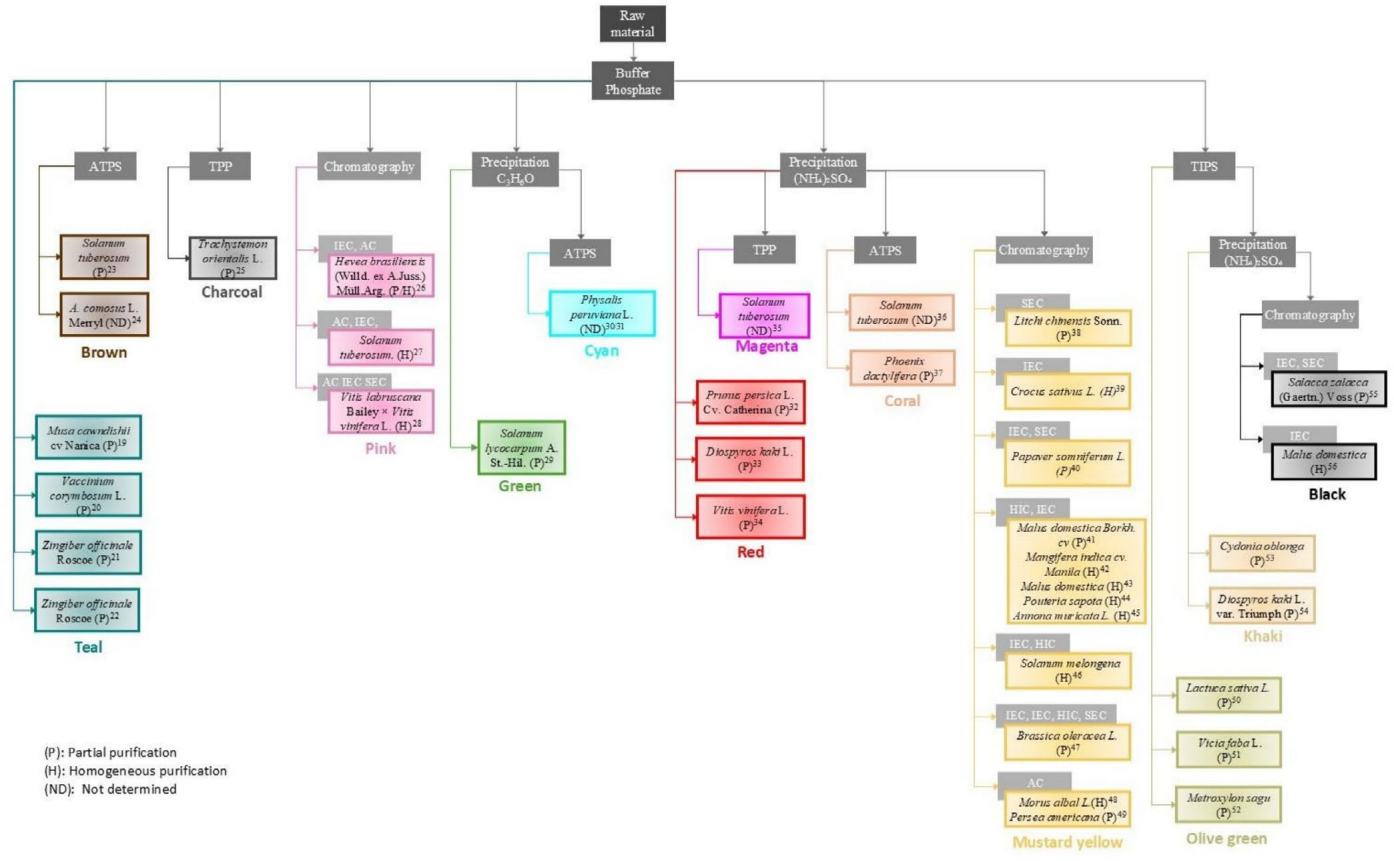
Purification train using sodium phosphate buffer. Teal *Musa cavendishii* ^19^ (Montgomery and Sgarbieri 1975; Wuyts et al. 2006), *Vaccinium corymbosum* L.^20^ (Siddiq and Dolan 2017), *Zingiber officinale* Roscoe^21^ (Lim and Wong 2018), *Solanum lycocarpum* ^22^ (Batista et al. 2014) achieved partial purification with Na phosphate buffer. Brown *Solanum tuberosum* ^23^ (Niphadkar and Rathod 2015; ATPS, PEG and 50 mM K phosphate) and *Ananas comosus* L. Merr.^24^ (Babu et al. 2008; ATPS, polyethylene glycol, 12%–18%, w/w, and K phosphate, 14%–20%, w/w). Charcoal *Trachystemon orientalis* L.^25^ (Alici and Arabaci 2016; TPP, saturated with 15% (w/v) ammonium sulfate and t‐butanol in the ratio of 1:1 v/v). Pink, *Hevea brasiliensis* (Willd. ex A. Juss.) Müll. Arg^26^ (Muhamad et al. 2012; IEC, DEAE‐Sepharose CL‐6B preequilibrated with 20 mM phosphate buffer pH 8.0, AC preequilibrated with 0.5 mM NaCl, 5 mM MgCl_2_, 5 mM MnCl_2_, and 5 mM CaCl_2_); *Solanum tuberosum* ^27^ (Bøjer Rasmussen et al. 2021; AC HiTrap Blue column preequilibrated with 25 mM phosphate, pH 7, IEC HiTrap Q HP column preequilibrated with 25 mM Tris–HCl, pH 8, IEC, HiTrap SP HP column preequilibrated with 25 mM sodium acetate, pH 5, SEC Sephacryl S‐200 preequilibrated with 25 mM sodium acetate and 150 mM NaCl, pH 5); *V. labruscana* Bailey × *V. vinifera* L.^28^ (Katayama‐Ikegami et al. 2017; AC nickel‐nitrilotriacetic acid‐agarose, IEC MonoQ 5/50 GL, SEC Superdex 200 10/300 GL preequilibrated with 20 mM Tris–HCl pH 8.5). Green, *Solanum lycocarpum* ^29^ (Batista et al. 2014; Crude extract was partially purified by freezing). The precipitate was homogenized in 50 mM Na phosphate buffer pH 6.5, containing 0.1% SDS (w/v). Cyan achieved partial purification PPO with ATPS from *Phaseolus peruviana* L.^30/31^ (Bravo and Osorio 2016; Bravo et al. 2011). Red *Prunus persica* L. Cv. Catherina^32^ (Cabanes et al. 2007; 25%–80% ammonium sulfate), *Diospyros kaki* L.^33^ (Navarro et al. 2014; 30%–85% ammonium sulfate) and *Vitis vinifera* L.^34^ (Lerner et al. 1972; 45%–95% ammonium sulfate). Magenta reached partial purification with TPP after 20%–80% ammonium sulfate of *Solanum tuberosum* PPO^35^ (Niphadkar and Rathod 2015). Coral got partial purification PPO with ATPS after 0%–25% ammonium sulfate of *Solanum tuberosum* ^36^ (Vaidya et al. 2006) and *P. dactylifera* ^37^ (Farouk et al. 2020) with 40%–80% ammonium sulfate and IEC (Q Sepharose Big Beads column (1.5 × 18 cm) previously equilibrated with 0.01 M Na phosphate buffer pH 7). Mustard Yellow has been the most proficient train (Na phosphate buffer precipitation with ammonium sulfate and chromatography). *Litchi chinensis* Sonn^38^ (Yue‐Ming et al. 1997; 50%–80% ammonium sulfate, SEC Sephadex G‐100 column preequilibrated with 0.01 M phosphate buffer (pH 6.8)), *Crocus sativus* L.^39^ (Esmaeili et al. 2017; 50%–80% ammonium sulfate, IEC equilibrated with 5 mM phosphate buffer pH 8.0), *Papaver sofniferum* L.^40^ (Hsu et al. 1984; 0%–85% of ammonium sulfate IEC carboxylmethylceulose equilibrated with 0.01 M Na phosphate buffer pH 6.8 containing 0.2 M sucrose, SEC Sephacryl S‐200 equilibrated with 0.05 M Na phosphate buffer pH 6.8 containing 0.2 M sucrose); *Malus domestica* Borkh. cv Bramley's Seedling^41^ (Ni Eidhin et al. 2006; 0%–85% ammonium sulfate), HIC, phenyl‐sepharose 4‐fast flow equilibrated with 50 mM sodium phosphate buffer, pH 7.0, containing 1.2 M ammonium sulfate and 1.2 M potassium chloride and IEC, Mono Q HR 5/5, with low‐ionic‐strength buffer (20 mM Tris–HCl, pH 7.0) followed by high‐ionic‐strength buffer (20 mM Tris–HCl, pH 7.0, containing 1.0 M potassium chloride) and finally with‐low‐ionic strength buffer (20 mM Tris–HCl pH 7.0); *Mangifera indica* cv. Manila^42^ (Palma‐Orozco et al. 2014; 30%–85% ammonium sulfate, HIC phenyl‐Sepharose equilibrated with 50 mM Na phosphate, pH 7.0, 1.2 M ammonium sulfate, and 0.6 M potassium chloride, SEC Mono Q HR 10/10 equilibrated with 20 mM TrisHCl buffer pH 7.0, containing 1.0 M KCl); *Malus domestica* ^43^ (Marrufo‐Hernández et al. 2017; 35%–85% ammonium sulfate, HIC phenyl‐Sepharose equilibrated with 50 mM Na phosphate, pH 7.0, 1.2 M ammonium sulfate, and 0.6 M potassium chloride, SEC Mono Q HR 10/10 equilibrated with 20 mM TrisHCl buffer pH 7.0); *Pouteria sapota* ^44^ (Palma‐Orozco et al. 2011; 30%–85% ammonium sulfate, HIC phenyl‐Sepharose equilibrated with 50 mM Na phosphate, pH 7.0, 1.2 M ammonium sulfate, and 0.6 M potassium chloride, SEC Mono Q HR 10/10 equilibrated with 20 mM TrisHCl buffer pH 7.0); *Annona muricata* L.^45^ (Palma‐Orozco et al. 2019; 30%–85% ammonium sulfate, HIC phenyl‐Sepharose equilibrated with 50 mM sodium phosphate, pH 7.0, 1.2 M ammonium sulfate, and 0.6 M potassium chloride, SEC Mono Q HR 10/10 equilibrated with 20 mM TrisHCl buffer pH 7.0); *Solanum melongena* ^46^ (Mishra et al. 2012; 50%–70% of ammonium sulfate, IEC DEAE (diethylaminoethyl) Cl‐6B Sepharose column equilibrated with 2 mM phosphate buffer pH 8.0, HIC phenyl Sepharose column equilibrated with 20 mM phosphate buffer pH 6.8, SEC Superdex 200 GE healthcare column equilibrated with phosphate buffer with 150 mM NaCl); *Brassica oleracea* L.^47^ (Rahman et al. 2012; 80% ammonium sulfate, IEC, DEAE‐Toyopearl 650‐M 0.01 M phosphate buffer pH 7.0—IEC, CM‐Sephadex C‐50 0.01 M phosphate buffer pH 7.0—HIC, butyl‐Toyopearl 650‐M 0.01 M phosphate buffer pH 7.0—SEC, Toyopearl HW 55‐s 0.1 M phosphate buffer pH 7.0); *Morus alba* L.^48^ (Arslan et al. 2004; uses ammonium sulfate for precipitation but does not stablish what is the percentage, then use AC Sepharose 4B‐L‐tyrosine‐p‐amino benzoic acid equilibrated with 5 mM phosphate buffer pH 5) and *Persea americana* ^49^ (Moeini Alishah et al. 2023; 0%–80% of ammonium sulfate, AC Sepharose 4B‐L‐tyrosine‐p‐amino benzoic acid equilibrated with 0.05 M, Na phosphate buffer pH 5). Olive achieved partial purification PPO with TIPS of *Lactuca sativa* L.^50^ (Chazarra et al. 1996); *Vicia faba* L.^51^ (Sánchez‐Ferrer et al. 1990) and *Metroxylon sagu* ^52^ (Onsa et al. 2000). Khaki *Cydonia oblonga* ^53^ (Orenes‐Piñero et al. 2006; 30%–75% ammonium sulfate) and *Diospyros kaki* L. var. Triumph^54^ (Núñez‐Delicado et al. 2003; 50%–75% ammonium sulfate). Finally, black *Salacca zalacca* (Gaertn.) Voss^55^ (Zaini et al. 2013; 40%–80% ammonium sulfate, IEC HiTrap Phenyl Sepharose High Performance column, was equilibrated 0.1 M K phosphate buffer and 1.0 M ammonium sulfate, pH 6.8, SEC Superdex 200 HR 10/30 column equilibrated with 50 mM K phosphate buffer (pH 6.8) containing 0.15 M NaCl), and *Malus domestica* ^56^ (Liu et al. 2015; 60%–80% ammonium sulfate), IEC DEAE Sepharose column equilibrated with 0.05 M Na phosphate buffer pH 6.8.

The sodium phosphate buffer is the most extensively used for PPO purification from plants. Some protocols purified the enzyme from the crude extract as in the cases of *Musa cavendishii* cv Nanica (Montgomery and Sgarbieri 1975), *Musa acuminata* Grande naine (Wuyts et al. 2006), *Vaccinium corymbosum* L. (Siddiq and Dolan 2017), *Zingiber officinale* Roscoe (Lim and Wong 2018), and *Solanum lycocarpum* (Liu et al. 2023), in which the PPO was partially purified (Figure 3 teal color).

An aqueous two‐phase system (ATPS) was applied using a phosphate and polyethylene glycol (PEG) partitioning to study polyphenol oxidase from *S. tuberosum* (Niphadkar and Rathod 2015; Niphadkar et al. 2015) and *Ananas comosus* L. Merryl (Babu et al. 2008). As a result, 3.7‐ and 2.7‐fold purifications, with yields of 77.8% and 90%, were obtained, respectively (Figure 3, brown).

A triphasic partitioning (TPP), using *t*‐butanol and ammonium sulfate was employed for the partial separation of *Trachystemon orientalis* L. PPO starting from the crude extract (Alici and Arabaci 2016). As a result, a 3.59‐fold purification and a yield of 68.75% were obtained (Figure 3, charcoal).

Using chromatography, PPO purifications were directly carried out from crude extract (Figure 3, pink). For example, PPO (70 kDa) from *Hevea brasiliensis* Willd. ex A. Juss. Müll. Arg cell suspension (Muhamad et al. 2012) was purified using IEC and SEC; *S. tuberosum* PPO (69 kDa; Bøjer Rasmussen et al. 2021) was used with IEC‐HIC (hydrophobic interaction chromatography)–SEC and *Brassica oleracea* L. PPO (60 kDa; Rahman et al. 2012) was successfully purified with IEC, IEC, HIC, and SEC. In a study on *Vitis labruscana* Bailey × *Vitis vinifera* L. (Katayama‐Ikegami et al. 2017), the PPO gene was identified to produce a recombinant enzyme that was purified using AC (Affinity chromatography)–IEC–SEC. Partial isolation of membrane‐bound and soluble PPO was achieved in *S. tuberosum* (Liu et al. 2023), revealing differences in their optimum temperature and biochemical parameters. With this information, it is inferred that the findings will help improve processing conditions to reduce potato browning.

Another purification option employed acetone as a precipitating agent, resulting in a 6‐fold purification for PPO from *Solanum lycocarpum* (Batista et al. 2014; Figure 3, green). Bravo and Osorio (2016) reported a partial purification of *Physalis peruviana* L. PPO using ATPS (Figure 3, cyan), achieving 4.65‐ and 169.4‐fold purifications, with yields of 74.29% and 13.9%, respectively (Bravo et al. 2011).

Polyphenol oxidases from different species were partially purified using differential ammonium sulfate precipitations (Figure 3, red). For example, the membrane‐bound PPO (pI 5.8) and the soluble PPO (pI 5.7) from *Prunus persica* L. Cv Catherina (Cabanes et al. 2007) were studied using 25%–80% ammonium sulfate saturation. In another study, the enzyme from *Diospyros kaki* L. (Navarro et al. 2014) was characterized following precipitation with 30%–80% ammonium sulfate saturation. In contrast, other authors used 45%–95% ammonium sulfate saturation to purify PPO from *V. vinifera* L. (Lerner et al. 1972; Sánchez‐Ferrer et al. 1988), and observed that it could be activated with urea, causing conformational changes.

In Figure 3 (Magenta), partial purification of the PPO from *S. tuberosum* can be observed. The initial step of its purification involved precipitation with acetone, followed by TPP, using different concentrations of ammonium sulfate and *t*‐butanol. In this case, a 6.3‐fold purification and a 70% recovery were obtained (Niphadkar and Rathod 2015). Similarly, using this method, *Lactuca sativa* L. PPO was partially purified, achieving a 5‐fold purification and a 70% yield (Chazarra et al. 1996).

PPO from *S. tuberosum* was partially purified using an ATPS with phosphate and polyethylene glycol (PEG), resulting in a 15.7‐fold purification and a yield of 97% (Vaidya et al. 2006; Figure 3).

Several PPO purified to homogeneity has been reported, with one or two chromatography steps (HIC‐IEC) following precipitation (Figure 3, mustard yellow), as seen in *Crocus sativus* L. (Esmaeili et al. 2017) and *Mangifera indica* cv. Manila (Palma‐Orozco et al. 2014), *Malus domestica* (Marrufo‐Hernández et al. 2017), *Pouteria sapota* (Palma‐Orozco et al. 2011), and *Annona muricata* L. (Palma‐Orozco et al. 2019), where this purification train stands out from the others. With this approach, a PPO partial purification has been obtained from *Litchi chinensis* Sonn (Yue‐Ming et al. 1997) and *Papaver somniferum* L. (Hsu et al. 1984).

Other authors have used temperature‐induced phase partitioning (TIPS) with Triton, where partial PPO purifications were obtained, for *Lactuca sativa* L. (5‐fold purification and 70% yield; Chazarra et al. 1996), *Vicia faba* L. (12.4‐fold purification and 43% yield; Sánchez‐Ferrer et al. 1990), and *Metroxylon sagu* (4.1‐fold purification and 69.82% yield; Onsa et al. 2000; Figure 3, olive green).

A combination of TIPS and ammonium sulfate precipitation of PPO from *Cydonia oblonga* (Orenes‐Piñero et al. 2006) and *Diospyros kaki* L. (Núñez‐Delicado et al. 2003) resulted in a 4.2‐ and 4.5‐fold purification and yields of 56.3% and 95%, respectively (Figure 3, khaki). Finally, chromatography was added as a separation step after TIPS of PPO from *Salacca zalacca* (Gaertn.) Voss (14.1‐fold purification and a yield of 12.3%; Zaini et al. 2013) and *Ma. domestica* Borkh. cv. Red Fuji (54.4‐fold purification and a yield of 0.05%; Liu et al. 2015; Figure 3, black).

Among the components used to obtain the crude extract, three are critical as follows: the polyvinylpolypyrrolidone (PVPP) eliminates the interactions of phenolic compounds with PPO; triton, mainly X‐100, allows the solubilization of PPO bound to the membrane without denaturation of proteins, and phenylmethylsulfonyl fluoride (PMSF) a serine protease inhibitor, which is used to maintain activity during the purification process; out of the total purification trains shown in figures 1–3, 20% utilized PMSF during the purification process (Iqbal et al. 2019; Mishra et al. 2012; Murtaza et al. 2019; Navarro et al. 2014; Ni Eidhin et al. 2006; Palma‐Orozco et al. 2019, 2011). It is worth noting that 64% of the reviewed articles use PVPP, and 50% use detergent to obtain the crude extract.

As previously mentioned, this review focuses on analyzing the methodologies used in plants, among which nine studies stand out due to their homogeneity (Table 1). This table shows the plants from which the PPO has been purified, the type of crude extract preparation used, the precipitating agent, the yield, and the fold purification. The pathway with higher PPO purity has utilized sodium phosphate buffer and ammonium sulfate as precipitating agents.

**TABLE 1:** PPO purification steps from different sources.

Raw material	Preparation type	Yield (%)	Purification (fold)
*N. tabacum* (Shi et al. 2002)	Acetone	22	100
*Ma. domestica* Borkh. (Quan et al. 2019)	McIlvaine	3	216
*Man. indica* cv. Manila (Palma‐Orozco et al. 2014)	Phosphate buffer	1.8	20.53
*C. sativus* L. (Esmaeili et al. 2017)	Phosphate buffer	ND	24.59
*Ma. domestica* (Marrufo‐Hernández et al. 2017)	Phosphate buffer	1.6	318.6
*Po. sapota* (Palma‐Orozco et al. 2011)	Phosphate buffer	11 *0.28	183 *6.6
*A. muricata* L. (Palma‐Orozco et al. 2019)	Phosphate buffer	3	160
*S. melongena* L. (Mishra et al. 2012)	Phosphate buffer	0.02	259
*B. oleracea* L. (Rahman et al. 2012)	Phosphate buffer	8.1	282

*Note:* This table shows the information on the study models in which the enzymes were purified to homogeneity. All these isolations use (NH_4_)_2_SO_4_. * are for isoenzymes.

Abbreviation: ND, not determined.

Chromatography methods, whether following crude extract precipitation or employed directly, have yielded promising results across various species, underscoring the adaptability and versatility of purification protocols. The applications of aqueous two‐phase systems and triphasic partitioning further illustrate the effectiveness of advanced separation techniques. These findings underscore the necessity for continued exploration and refinement of PPO purification techniques to enhance enzyme yield and activity, paving the way for future studies on enzyme kinetics and their potential applications in food processing and other industries. Overall, the results emphasize the complexity of PPO purification across different species and the potential of various methodologies to enhance product purity and enzyme characterization.

### 2.3 Chromatographies

Figure 1 illustrates the various chromatographies employed on crude extracts obtained using acetone. These purifications cannot be easily compared owing to the fold purification and the yield not being reported. The most commonly used chromatographies in these protocols are IEC and SEC. There are different ways to combine these chromatographies, but the IEC–SEC–IEC combination allowed the enzyme to be purified to homogeneity (yellow train). In the latter, a 100‐fold purification and a yield of 22% were obtained from *N. tabacum* (Shi et al. 2002).

At the buffer pathway, the most used chromatography was IEC (Figure 2). Different methods for combining chromatographies were reported; the one that proved optimal was IEC–SEC. With this combination, a pure enzyme from *Ma. domestica* Borkh was obtained, with a 20.5‐fold purification and a yield of 1.8% (Han et al. 2020). In *V. planifolia* , homogeneous purification was achieved using SEC and isoelectric focusing, resulting in a 2.8‐fold purification and a yield of 1.1% (Waliszewski et al. 2009). When homogeneous purifications were achieved, better fold purifications were obtained; however, lower yields were observed (Table 1).

As in the other purification trains, when phosphate buffers were used, the most employed chromatography was IEC (Figure 3). There are various possibilities for combining chromatographies, some of which include the use of HIC. The most effective combination was HIC–IEC, which was applied to five plant species, and homogeneity was achieved in all of them. In *A. muricata* L. (Palma‐Orozco et al. 2019), *Man. indica* cv. Manila (Palma‐Orozco et al. 2014), *Ma. domestica* (Marrufo‐Hernández et al. 2017), *Solanum melongena* (Mishra et al. 2012), *H. brasiliensis* (Willd. ex A. Juss.) Müll. Arg (Muhamad et al. 2012), fold‐purification values of 160, 216, 318.6, 259, and 104.2, respectively, were obtained with corresponding yields of 3%, 3%, 1.6%, 0.02%, and 54.4%. The latter species yielded the best results (Table 1).

### 2.4 Affinity Columns

Despite the attempts, not in all cases was a purification to homogeneity obtained. Some years ago, Arslan et al. (2004), were the first to use affinity chromatography, obtaining a PPO from *Morus alba* L. with a 74‐fold purification using a Sepharose 4B‐L‐tyrosine‐*p*‐aminobenzoic acid column. Recently, new approaches in the PPO purification from *S. tuberosum* have been discussed, and some affinity columns have been developed. These columns consisted of 4‐aminophenol, 2‐aminophenol spacers, and L‐tyrosine spacer arms, with fold purifications of 12, 7, and 5, and yields of 2.39%, 0.89%, and 0.89%, respectively. The best affinity purification method was achieved with a 4‐aminophenol spacer arm (Aksoy 2020).

## 3 PPO Enzymatic Characterization

### 3.1 Biochemistry

In this section, the biochemical characterization of PPO since 1970 will be discussed. There are differences between PPO; these can be identified by their physical, chemical, or enzymatic properties, such as electrophoretic mobility, optimal temperature, optimal pH, MW, substrates, and inhibition (Lerner et al. 1972).

### 3.2 Optimum pH

The optimal activity pH range for PPO was found to be between 3.4 and 8. For instance, in *V. planifolia*, the PPO has the lowest optimum pH, and it is reported to function in acidic environments, such as the vacuole (Waliszewski et al. 2009). Interestingly, in tropical plants, a correlation was observed between the optimal temperature of the enzyme and the environments in which these plants thrive, with the optimum pH often being close to neutral pH (Das et al. 1997). In contrast, *Cleome gynandra* L. PPO has a basic optimal pH (Gao et al. 2011). This characteristic could be beneficial, as acidic solutions may act as enzyme inhibitors. For example, in the context of the enzymatic browning of *C. gynandra* L., the browning process can be controlled using acidic solutions (Gao et al. 2011).

### 3.3 pH and Enzyme Stability

The activity and stability of potato PPO vary depending on the pH of its environment. This study demonstrated that PPO exhibits greater stability at a pH of 7, and it maintains good stability within a pH range of 5.5 to 7.5, retaining approximately 80% of its activity (Niphadkar et al. 2015). Additionally, the stability of PPO was also observed in apricot, apple, eggplant, and potato species. The findings indicate that PPO remains stable within a pH range of 5.5 to 8, maintaining at least 80% of its relative activity (Mahmood et al. 2009). Overall, polyphenol oxidase is most stable at neutral pH levels, highlighting the importance of pH in regulating the activity and stability of PPO.

### 3.4 Optimum Temperature

The optimal temperature range for PPO activity is from 5°C to 70°C. Notably, the optimal temperature of *T. orientalis* L. PPO is the lowest reported, using 4‐methylcatechol as a substrate (Alici and Arabaci 2016). Most plant PPOs studied to date exhibit optimal temperature ranges between 20°C and 50°C. However, there are a few exceptions with optimal temperatures exceeding 50°C; for instance, the PPO from *C. gynandra* L., which has a high optimal temperature of 60°C (Gao et al. 2009). It is important to note that the optimal temperature is significantly influenced by the environment in which the study organism grows (Yoruk and Marshall 2003).

### 3.5 *K*_M_

The *K*_M_ values allow us to know the affinity of the PPOs for the analyzed substrates. Enzyme affinity for the substrate will largely depend on the enzyme source, the substrate structure, the extraction conditions, and the phenolic compounds found in the fruit from which the enzyme was purified. A variety of substrates have been used to measure PPO activity, with 4‐methylcatechol and catechol being the most commonly used. Table 2 illustrates the differences in *K*_M_ values, highlighting the affinity of PPO from different species for these substrates. When using 4‐methylcatechol as the substrate, the PPO with the highest affinity was obtained from *S. lycocarpum* (Batista et al. 2014), while the lowest affinity was found in *Ph. peruviana* (Bravo and Osorio 2016). For catechol, the PPO with the best affinity came from *H. brasiliensis* (Wuyts et al. 2006), whereas *V. planifolia* showed thelowest affinity (Waliszewski et al. 2009). The structures of 4‐methylcatechol and catechol are quite similar, with the only difference being the presence of a methyl group in 4‐methylcatechol (Batista et al. 2014). The PPO's affinity for the presence or absence of the methyl group varies by species; however, in all cases, higher *K*_M_ values for 4‐methylcatechol correspond to lower *K*_M_ values for catechol, and vice versa.

**TABLE 2:** Comparative *K*_M_ values PPO from different sources.

Source	4‐metylcatechol *K*_M_ (mM)	Catechol *K*_M_ (mM)
*S. lycocarpum* (Batista et al. 2014)	0.15	6.47
*A. muricata* L. (Palma‐Orozco et al. 2019)	0.86	3.16
*P. americana* Mill. Fuerte (Kahn and Pomerantz 1980)	0.9	ND
*Ma. domestica* (Marrufo‐Hernández et al. 2017)	1.3	7.5
*P. americana* Mill. Fuerte (Dizik and Kanapp 1970)	2	6.8
*T. orientalis* L. (Alici and Arabaci 2016)	4.55	2.24
*Ma. domestica* Borkh. (Han et al. 2020)	7.31	11.92
*M. cavendishii* cv Nanica (Wuyts et al. 2006)	8.3	69
*V. vinifera* L. (Lerner et al. 1972)	9	ND
*Litchi chinensis* Sonn. (Yue‐Ming et al. 1997)	10	ND
*V. planifolia* (Waliszewski et al. 2009)	10.6	85
*Pr. armeniaca* L. (Derardja et al. 2017)	11	5.3
*Va. corymbosum* L. (Siddiq and Dolan 2017)	15	ND
*Man. indica* cv. Manila (Palma‐Orozco et al. 2014)	15	2.7
*Prunus domestica* (Ioniţă et al. 2017)	15.5	26.3
*Ph. peruviana* L. (Bravo and Osorio 2016)	203.8	3.88
*S. melongena* L. (Roudsari et al. 1981)	222	7
*S. melongena* L. (Mishra et al. 2012)	0.34	ND
*P. americana* Mill (Gómez‐López 2002)	0.73 0.58	10.4 9.24
*Pho. dactylifera* L. (Farouk et al. 2020)	1.9	ND
*Papaver somniferum* (Hsu et al. 1984)	ND	5
*Ca. sinensis* (Halder et al. 1998)	ND	12.52
*N. tabacum* (Shi et al. 2002)	ND	1.2
*D. kaki* L. (Navarro et al. 2014)	ND	25
*Po. sapota* (Palma‐Orozco et al. 2011)	ND	44
*H. brasiliensis* (Muhamad et al. 2012)	ND	0.125
*Man. indica* (Cheema and Sommerhalter 2015)	ND	0.41
*Ca. sinensis* (Teng et al. 2017)	ND	23.00

*Note:* This table shows information about *K*_M_ with two different reference substrates.

### 3.6 MW

There is a wide variety of reported molecular weights for PPO, ranging from 14 to 400 kDa. Studies have shown that approximately 85% of PPOs have molecular weights between 14 and 100 kDa, while 10% fall within the range of 101 and 200 kDa, and the remaining 5% exceed 201 kDa. It is important to note that different methods have been employed to determine the molecular weight of the enzyme, including SDS‐PAGE, gel filtration, and MALDI‐TOF‐MS. For instance, in the case of *P. sapota* , PPOs with molecular weights of 16.1 and 18 kDa were identified using gel filtration and SDS‐PAGE, respectively (Palma‐Orozco et al. 2011). In contrast, *Pho. dactylifera* exhibited molecular weights of 20.45 and 64 kDa, as determined through SDS‐PAGE (Farouk et al. 2020).

Additionally, some studies have reported the presence of up to five isoforms of PPO within the same model organism. For example, *P. americana* Mill. has been shown to possess five isoforms with molecular weights ranging from 14 to 400 kDa (Dizik and Kanapp 1970).

## 4 Inhibition

### 4.1 Chemical Inhibition

Chemical inhibition of PPO activity can be achieved through several methods. One effective approach is acidification, which involves lowering the pH using substances like citric acid or ascorbic acid. This reduction in pH leads to decreased PPO activity. Additionally, antioxidants such as ascorbic acid, glutathione, and L‐cysteine can impede the browning process by reacting with the intermediates produced by PPO. Another method is the use of chelating agents like citric acid and oxalic acid, which bind to the copper ions within the PPO enzyme, thereby reducing its activity. Lastly, sulfur‐containing compounds, including potassium metabisulfite, can also inhibit PPO activity (Han et al. 2021; Yaseen 2022; Liao et al. 2020, 2021; Liu et al. 2024).

### 4.2 Physical Inhibition

Physical inhibition is a method used to manage enzymatic activities in food products. Heat treatment is a common thermal inactivation technique; however, it can negatively impact the quality of the food. Non‐thermal methods such as ultrasound, high hydrostatic pressure, and pulsed light can effectively disrupt an enzyme's structure and reduce its activity. Additionally, using oxygen‐impermeable packaging can significantly decrease oxygen contact, which helps inhibit browning. Certain natural extracts, such as chicory furaneol and honey, have shown inhibitory effects on polyphenol oxidase (PPO). In particular, chicory furaneol has been identified as a natural PPO inhibitor, demonstrating strong effects that can extend the shelf life of fresh‐cut potatoes. Furthermore, employing combined approaches that integrate both physical and chemical methods can be more effective in inhibiting PPO and preventing browning in food products (Derardja et al. 2019; Iqbal et al. 2019; Liang et al. 2024).

The methods that have shown better inhibition efficacy include cold plasma, thermosonication, and treatment with superheated steam (Table 3). Notably, research conducted on soursop nectar revealed that thermosonication effectively inhibits PPO without damaging the nutritional properties of the product (Guo et al. 2020).

**TABLE 3:** Novel PPO inhibitors. This table presents some of the most effective methods for inhibiting polyphenol oxidase in various species.

Inhibitor	Inhibition (%)	Condition
Chitosan (Xing et al. 2011)	ND	0.5%, 0.7%, and 1%
Ultrasound and ascorbic acid (Jang and Moon 2011)	15	Frequency 40 kHz, ascorbic acid 1%
Eugenol (Huang et al. 2019)	30	25 μL/L
Strawberry by‐products rich in phenolic compounds (Villamil‐Galindo et al. 2020)	30	0.25 mL (strawberry by‐product polyphenol extract)
B‐cyclodextrin (Singh et al. 2015)	40	17.6 mM
Some organic acids (Liao et al. 2020)	50	Ascorbic acid (3, 6 and 12 mmol/L), citric acid (30, 60,120, and 148 mmol/L), salicylic acid (10, 20 and 30 mmol/L)
Superheated steam treatment (Guo et al. 2020)	83	155°C–170°C
Detergents (Saeidian et al. 2007)	20–92	0.03–150 mM
Thermosonication (Anaya‐Esparza et al. 2017)	67–99	Acoustic energy density 1.1–1.4
Cold plasma (Illera et al. 2019)	100	Spark discharge plasma at 10.5 kV

### 4.3 Mechanism of PPO Inhibition

The mechanism of PPO inhibition involves several processes. Competitive inhibition occurs when some inhibitors compete with oxygen or phenolic compounds for the active site of the enzyme. Structural changes can occur through physical treatments, such as ultrasound and ultraviolet radiation, which can disrupt the enzyme's structure and reduce its activity. Additionally, copper chelation involves chelating agents that bind to the copper ions within the PPO enzyme, rendering it inactive. Furthermore, some inhibitors, such as ascorbic acid, exhibit antioxidant activity that prevents the oxidation of phenolic compounds (Cheng et al. 2025; Liang et al. 2024). The significance of inhibiting polyphenol oxidase (PPO) is evident in several key areas. First, preventing enzymatic browning is crucial for maintaining the color, flavor, and nutritional quality of fruits and vegetables. Second, inhibiting PPO can extend the shelf life of fresh‐cut produce and various processed foods. Additionally, the inhibition of PPO is significant for minimally processed items, such as fresh‐cut fruits and vegetables (Liang et al. 2024; Liu et al. 2024).

## 5 Structural Studies

### 5.1 Intrinsic Fluorescence

The fluorescence study offers insights into the conformational changes that occur in the tertiary structure of enzymes. These analyses could be extended by comparing how environmental conditions—such as temperature, pH, and the presence of inhibitors—affect the structural modifications of the native state. In terms of PPO fluorescence emissions under native conditions, they have been reported for the enzyme from *Ma. domestica*: 311 (Murtaza et al. 2019), 315 (Han et al. 2020, 2021), and 331 (Iqbal et al. 2018) nm. In contrast, the PPO from *C. oblonga* Miller exhibited a λ_max_ of 340 nm, attributed to the greater exposure of aromatic amino acids. Furthermore, in PPO from *Ma. domestica* a red shift in fluorescence was observed when using high‐pressure carbon dioxide (HP‐CO_2_), as this type of inhibition led to an increased exposure of aromatic amino acids (Murtaza et al. 2019).

The finding of Han et al. (2021) is novel and exciting, as they observed a significant decrease in PPO fluorescence intensity in the presence of four inhibitors (L‐cysteine, glutathione, ascorbic, and citric acids). The most potent inhibitor, ascorbic acid, was identified as a competitive inhibitor, demonstrating a high affinity for the enzyme. This finding is particularly interesting, as it was supported by the inhibitor's higher fluorescence intensity quenching, which resulted from a conformational change.

Another example of the use of fluorescence was the study of the inhibition mechanism of cyanidin‐3‐sophoroside (cs). This inhibitor binds to PPO, affecting the proximity of the fluorophore microenvironment and thus quenching the intrinsic fluorescence intensity of the enzyme (Hemachandran et al. 2017). The study of PPO fluorescence has proven highly useful in elucidating how environmental changes affect the tertiary structure of the enzyme. This avenue of research should be further explored because it can provide essential information.

### 5.2 Circular Dichroism

Circular dichroism studies are key to determining the protein's secondary structures and their percentage (Table 4). They also play a crucial role in understanding the conformational changes that occur with variations in the environmental conditions of the enzyme, either due to temperature changes or the use of inhibitors (Onsa et al. 2000). For example, circular dichroism has been used to determine the inhibition mechanism of PPO by cyanidin‐3‐sophoroside. It was found that the percentage of α‐helix and β‐sheet structures after PPO interaction with cs caused a relaxation of the native structure. This conformational change was the result of the orientation of the amino acid residues, which allowed for a low energy level and thus maintained the stability of the polypeptide chain (Hemachandran et al. 2017).

**TABLE 4:** Secondary structure percentages obtained from the deconvolution of circular dichroism spectra of the PPO from different sources.

Source	α‐helix (%)	β‐sheet (%)	β‐turn (%)	Random coil (%)
*Ma. domestica* (Murtaza et al. 2019)	23.56	40.81	18.41	17.22
*Ma. domestica* (Hemachandran et al. 2017)	24.9	17.7	22	35.4
*Ma. domestica* Borkh. (Han et al. 2020)	36.4	4.9	26.1	12.9
*N. tobacum* (Shi et al. 2002)	26.3	25.9	17	29
*Dolichos lablab* (Kanade et al. 2006)	29	ND	ND	ND
*Cy. oblonga* Miller (Iqbal et al. 2018)	39.34	17.9	26.41	16.35
*S. tuberosum* Jasim (Jang and Song 2004)	35	0	30	35

*Note:* This table displays some of the data reported on the secondary structure using circular dichroism, spanning the wavelength range from 250 to 180 nm.

On the other hand, the effect of high‐pressure carbon dioxide on PPO has been studied, showing that this treatment causes a decrease in the α‐helix and an increase in β‐sheet percentages, resulting in a reduction of enzymatic activity (Murtaza et al. 2019). In three of these studies (Han et al. 2020; Iqbal et al. 2018; Jang and Song 2004), PPO composition consists mainly of α‐helix, so it has been reported that this high content forms the helical bundle, causing the active site of the PPO to be accommodated (Table 4). It is necessary to accumulate more information of this nature to obtain more comprehensive PPO structural information.

### 5.3 Comparative Studies of PPO Sequence and Structure

PPO, being a copper‐containing enzyme, catalyzes two key reactions involving molecular oxygen and various phenolic substrates. It first converts monophenols into o‐diphenols (cresolase activity) and then oxidizes o‐diphenols into o‐quinones (catecholase activity). This process ultimately leads to the formation of melanins through polymerization. During melanogenesis from L‐tyrosine, PPO converts it into L‐DOPA, which then transforms into o‐dopaquinone. This compound further converts into dopachrome and ultimately polymerizes to form melanin. PPO can also act directly on o‐diphenolic substrates, bypassing the initial step of converting monophenols to o‐diphenols (Faria et al. 2007; Tilley et al. 2023).

**FIGURE 4:**
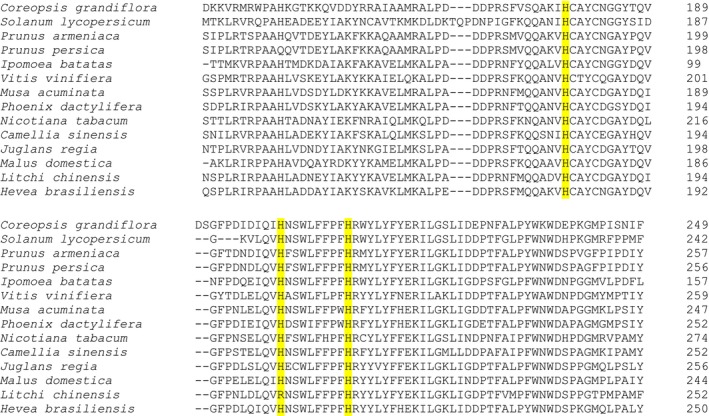
Partial sequence alignment where are shown highlighted histidines (yellow) are binding sites to CuA.

Using UniProt, an amino acid sequences alignment was done (Figure 4); as a result, it was found that among different fruit PPOs, copper‐binding sites, (which are the conserved histidines as mentioned above) are in the same range, that is, although the histidines are in different positions, the distance from one to the other is specific and conserved.

**FIGURE 5:**
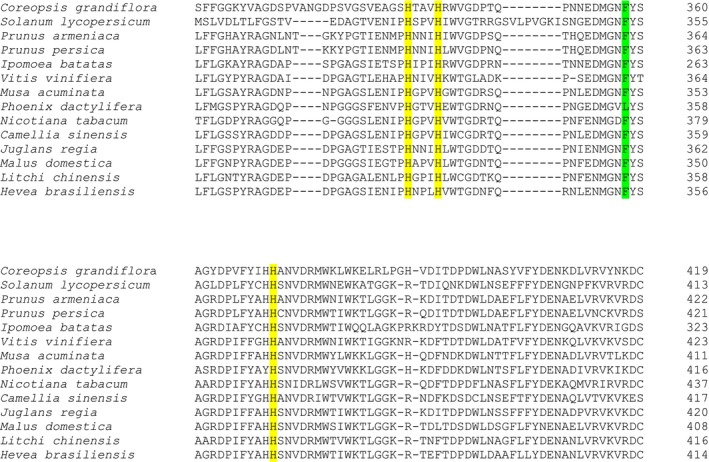
Partial sequence alignment where highlighted histidines (yellow) and phenylalanine gate‐residue (green) that interact with Cu_B_.

With comparative bioinformatics studies, it is possible to infer the general structural features of the polyphenol oxidases. This structure consists of three main regions: the N‐terminal, the catalytic active core, and the C‐terminal. Some of the components can be readily observed in the alignment (Figures 4 and 5), such as the N‐terminal, which is a chloroplast transit peptide and is of utmost importance to know the location to which the enzyme should be directed (Molitor et al. 2015, 2016). Another component that can be observed in the alignment is the tyrosinase domain, formed by a series of conserved histidines (three for CuA and three for CuB), as shown in Figures 4 and 5.

Molitor et al. (2016) stated the importance of the phenylalanine found at the binding site for CuB; this residue can be observed in the alignment sequences, as seen in Figure 5. The reported function of this amino acid is similar to a gate, since it is located above the active site of PPO. It should be noted that other residues that function as a small plug have also been found; these residues are leucine, valine, isoleucine. Also of note is a conserved PPO motif, which is a cysteine residue that forms a thioether bridge with one of the CuA‐binding histidines. Kaintz et al. (2014), proposed that both the phenylalanine residue and the cysteine are necessary for the substrate to be wholly bound and in the appropriate orientation.

In the crystallized PPOs from *Juglans regia* , *Ipomoea batatas* , and *V. vinifera* , the previously mentioned blocking residues are found on the CuA binding site. The latter has been associated mainly with catechol oxidases, so it was proposed that since the substrates could not interact with CuA, the enzyme would lack monophenolase activity and that in turn, if the residues were on CuB, it would lack catechol oxidase activity (Decker et al. 2006). It should be noted that the term “blocker” was proposed some years ago for reconsideration as a description of this residue's function, given a study that demonstrated its flexibility, which allows access to the substrate at the active site (Bijelic et al. 2015; Fronk et al. 2015).

**FIGURE 6:**
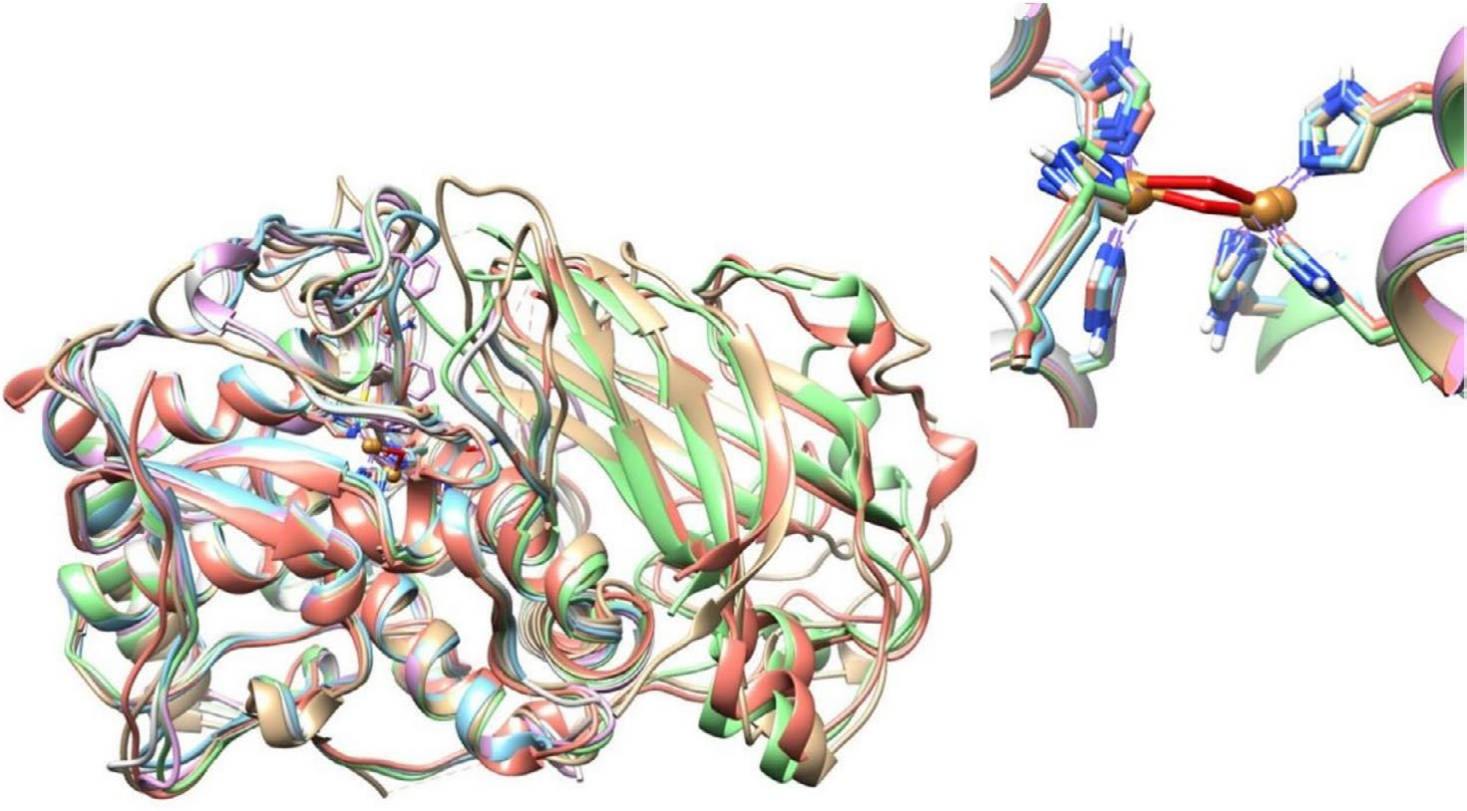
Structural alignment from crystallized PPOs and deposited in the PDB. Some structural differences can be observed, mainly in loops and α‐helix. PPO colors are as follows: Gold, *Coreopsis grandiflora* ; cyan, *Ipomoea batatas* ; purple, *Juglans regia* ; green, *Malus domestica* ; red, *Solanum lycopersicum* , and gray *Vitis vinifera* . Inset: Binding site of cupper.

Crystallized structures of PPOs are limited, restricting our understanding of the differences among them. The available structures include from *I. batatas* (PDB 1BT3), *V. vinifera* (PDB 2P3X), *Coreopsis grandiflora* (PDB 4Z11), *J. regia* (PDB 5 ce9), *Ma. domestica* (PDB 6ELS), and *S. lycopersicum* (PDB 6HQI; Table 5). The last two PPOs listed allow for the visualization of their C‐terminal domains (Figure 6).

**TABLE 5:** Crystallization parameters of different PPOs.

Source	Conditions of crystallization	Crystals	Space group	Resolution (Å)	PDB code
*I. batatas* (L.) Lam. (Klabunde et al. 1998)	PEG 6000, 500 mM NaCl, 50 mM Hepes, pH 7.0 equilibrated against a solution containing 200 mg ml^−1^ PEG 6000 By hanging‐drop vapor diffusion	Monoclinic Orthorhombic	P2_1_ P2_1_2_1_2	2.50	1BT3
*V. vinifera* (Virador et al. 2010)	Citrate buffer, pH 5.6, were mixed with the reservoir solution that had 30% (w/v) PEG‐4000. Buffer citrate pH 5.6 and 200 mM ammonium acetate Hanging‐drop vapor‐diffusion	Orthorhombic	*C*222_1_	2.20	2P3X
*Co. Grandiflora* (Molitor et al. 2016)	0.2 *M* ammonium acetate, 0.1 *M* sodium citrate pH 5.6, 30% PEG 4000 Hanging‐drop vapor‐diffusion	Orthorhombic	P12_1_1 P2_1_2_1_2_1_	2.50	4Z11
*J. regia* (Bijelic et al. 2015)	Reservoir solution consisting of 30% PEG 5000 in monomethyl ether, 200 mM ammonium sulfate and 100 mM MES pH 6.5. Hanging drop vapor‐diffusion	Monoclinic	C121	2.39	5 CE9
*Ma. domestica* (Kampatsikas et al. 2017)	50 mM Tris–HCl pH 7.0, 19%–21% PEG 3350 Hanging drop vapor‐diffusion	Orthorhombic	P2_1_2_1_2_1_	1.346	6ELS
*S. lycopersicum* (Kampatsikas et al. 2019)	50 mM sodium citrate pH 6.8, 13% w/v PEG 8000 Hanging drop vapor‐diffusion	Orthorhombic	P1211	1.85	6HQI

*Note:* This table displays the crystallization values of different plant polyphenol oxidases crystallized thus far.

It was reported that the Aurone synthase structure from *Co. grandiflora* is highly similar to catechol oxidases found in *I. batatas* , *V. vinifera* , and *J. regia* , having a 47% sequence identity with catechol oxidases and 48% identity with tyrosinases (Molitor et al. 2016). A structural alignment of the crystallized structures, references *V. vinifera* 's PPO. The identity percentages from amino acid sequences were 63% ( *J. regia* ), 63.6% (*Ma. domestica*), 57.9% ( *I. batatas* ), 47.8% (*Co. grandiflora*) and 46% ( *S. lycopersicum* ).

The identity sequence using *V. vinifera* as reference was as follows: 62.5% ( *J. regia* ), 57.5% (*Ma. domestica*), 57.4% ( *I. batatas* ), 49% (*Co. grandiflora*), and 41.9% ( *S. lycopersicum* ). The main differences among structures were found in the α‐helices and the loops.

Table 5 shows some of the crystal parameters of the PPOs found in the PDB. Aurone synthase (found in *Co. grandiflora*) crystallized in space groups P12_1_1 and P2_1_2_1_2_1_, formed by four monomers and crystallized at a resolution of 2.50. The structure of aurone synthase is highly similar to PPOs from *I. batatas* , with which it has an RMSD of 0.81 Å, *V. vinifera* , with an RMSD of 0.79 Å, and *J. regia* , with an RMSD of 0.82 Å (Kanade et al. 2006).

Regarding the crystallization conditions, it can be observed that these crystals were obtained using similar methodologies (Table 5), in which PEG, ammonium acetate, and sodium citrate are the most commonly used components (Kampatsikas et al. 2019; Klabunde et al. 1998; Molitor et al. 2015; Virador et al. 2010; Zekiri et al. 2014). In four of the six structures mentioned above, hanging‐drop vapor‐diffusion methodologies have been used, except for two of them ( *J. regia* and *Ma. domestica*). In the latter two, crystallizations were initiated by sitting‐drop vapor‐diffusion, and later, the optimization was done by hanging‐drop vapor‐diffusion. The best resolution obtained so far was for *Ma. domestica* PPO (Kampatsikas et al. 2017, 2019).

### 5.4 Applications

Due to the functionalities of polyphenols, the conjugation of proteins/polyphenols is becoming an important focus of research, compared to other protein‐polymer conjugates (Quan et al. 2019). Polyphenol oxidase has been very useful in the food industry, where the protein‐polyphenol conjugates can be used as novel food additives for the improvement of functionalities and quality of food products (Quan et al. 2019), in the development of biosensors for the identification of phenolic compounds in industrial sources such as petrochemicals, paints, dyes, textiles, pharmaceuticals, and plastics (Mu'azu et al. 2017). Immobilization polymerization allows the formation of insoluble aggregates in water, which are removed by sedimentation or filtration (Wang et al. 2020; Table 6).

**TABLE 6:** Applications of polyphenol oxidase.

Industry	Application
Food	In this industry it was found that the interaction of protein‐polyphenol conjugates have strong antioxidant effects and can work for food products (Quan et al. 2019)
Biosensor	This method works for detecting polyphenols, the biosensors are manufactured with polyphenol oxidase in the active layer (Li et al. 2010; Raymundo‐Pereira et al. 2020; Sartori et al. 2011)
Immobilization	These types of applications help to improve both the storage and the availability of the enzymes that are immobilized (Gür et al. 2019)
Elimination	The common use of polyphenol oxidase is in water treatment because PPO can catalyze a great variety of substrates, used to remove aromatic pollutant (Altinkaynak et al. 2018; Mukherjee et al. 2013)

## 6 Conclusion

Polyphenol oxidase is one of the most widely studied enzymes in terms of its sources and characterization. Numerous studies have investigated its extraction and purification from various plant species, proposing diverse strategies to optimize these processes.

This review presents a comprehensive compilation of such studies, highlighting that although the most commonly reported methods include salt precipitation, chromatography, TIPS, TPP, and ATPS, the most widely used strategy to achieve homogeneous PPO involves the use of sodium phosphate buffer, followed by a precipitation step and, ultimately, chromatographic techniques. The reviewed studies indicate that only chromatographic methods have successfully purified PPO to a homogeneous state.

The enzyme's kinetic and biochemical properties are essential for its characterization, as they enable the determination of substrate affinity, as well as mechanisms of action and inactivation. However, these properties can be significantly affected by extrinsic factors such as extraction conditions, pH, temperature, and others.

Furthermore, physicochemical techniques such as fluorescence and circular dichroism have proven to be valuable tools for studying the structure, conformational changes, and environmental influences on PPO—key aspects for a deeper understanding of its mechanism of action and potential applications. Nonetheless, these methodologies remain largely underexplored in this context.

Overall, this review compiles the current methods for extracting and purifying PPO from various plant sources, emphasizing the influence of both intrinsic and extrinsic factors on its biochemical and molecular characterization. Over time, the production of PPO from a broader range of plant species, with greater levels of purity, has increased—enabling more precise biochemical and structural studies. These advances have significantly enhanced our understanding of PPO activity and function; however, the practical application of this knowledge in the agri‐food industry remains a considerable challenge.

## Author Contributions

**Cesar G. Vazquez‐Lima:** conceptualization (equal), data curation (equal), formal analysis (equal), investigation (equal), methodology (equal), software (equal), validation (equal), visualization (equal), writing – original draft (equal), writing – review and editing (equal). **Mariana Quintana‐Quirino:** formal analysis (supporting), investigation (supporting), methodology (supporting), software (supporting), visualization (supporting), writing – review and editing (supporting). **Ana Luisa Bravo:** conceptualization (equal), formal analysis (supporting), investigation (supporting), methodology (supporting), software (supporting), supervision (supporting), visualization (supporting), writing – original draft (supporting), writing – review and editing (supporting). **Roxana López‐Simeon:** conceptualization (equal), formal analysis (supporting), investigation (supporting), methodology (supporting), software (supporting), supervision (supporting), visualization (supporting), writing – original draft (supporting), writing – review and editing (supporting). **Hugo Nájera:** conceptualization (lead), data curation (lead), formal analysis (lead), funding acquisition (lead), investigation (lead), methodology (lead), project administration (lead), resources (lead), software (lead), supervision (lead), validation (lead), visualization (lead), writing – original draft (lead), writing – review and editing (lead).

## Conflicts of Interest

The authors declare no conflicts of interest.

## Data Availability

Data openly available in a public repository that issues datasets with DOIs.
